# Breathless encounters: Analyzing anaphylaxis at the crossroads of COVID-19 vaccination

**DOI:** 10.5339/qmj.2024.qitc.9

**Published:** 2024-03-24

**Authors:** Pradipta Paul, Emmad Janjua, Mai AlSubaie, Vinutha Ramadorai, Beshr Mushannen, Ahamed Lazim Vattoth, Wafa Khan, Khalifa Bshesh, Areej Nauman, Ibrahim Mohammed, Imane Bouhali, Mohammed Khalid, Dalia Zakaria

**Affiliations:** 1 Weill Cornell Medicine–Qatar, Qatar Foundation, Education City, PO Box 24144, Doha, Qatar Email: prp4005@qatar-med.cornell.edu; 2 Department of Medicine, The Johns Hopkins Hospital, Baltimore, Maryland, USA; 3 Department of Internal Medicine, Albany Medical Center Hospital, Albany, New York, USA

**Keywords:** coronavirus, allergy, vaccine, inflammation, immunology

## Introduction

The COVID-19 pandemic necessitated the swift development and widespread distribution of vaccines. However, adverse events, including anaphylaxis, have been reported post-vaccination. While anaphylaxis remains a rare occurrence with an incidence of 1.31 in 1 million doses, our objective was to systematically review and analyze the available data on anaphylactic reactions following COVID-19 vaccination. The aim was to enhance patient safety and provide insights for clinicians to identify potential risk factors associated with patients or vaccines.^1,2^

## Methods

A systematic review included major scientific databases and examined records published in English until April 2022. Inclusion criteria focused on anaphylaxis or anaphylactoid reactions following COVID-19 vaccination, as verified by the Brighton Collaboration Criteria, ignoring demographic characteristics. Covidence facilitated screening, extraction, and quality assessment, which were conducted by at least two independent reviewers.

## Results

Of 19,908 initial records, 41 studies from 14 countries met the criteria, including six that used data from the United States Vaccine Adverse Event Reporting System (VAERS). Among the 7,942 reported anaphylaxis cases, Pfizer accounted for 66.5%, Moderna 13.5%, AstraZeneca 18.1%, Janssen 1.5%, Sinovac 0.2%, and unspecified mRNA vaccines < 0.1%. Most cases involved females with a documented history of allergies. Most cases occurred within 30 minutes of the first dose, with 43 confirmed deaths reported. Epinephrine was the primary treatment, often combined with other interventions. The rates of anaphylaxis per million doses administered ranged from 2.34 to 11.1 for Pfizer, 2.5 to 8.23 for Moderna, and 7.05 to 7.43 for Janssen.

## Conclusion

The incidence of anaphylaxis following COVID-19 vaccination slightly surpasses general vaccination rates, emphasizing the importance of careful screening. Healthcare professionals should thoroughly investigate and document allergic histories, administer vaccines in managed settings, extend observation periods for high-risk patients, and consider changing vaccine types in cases of severe allergic reactions. Referral to allergy–immunology specialists may be necessary for further guidance and care. These recommendations aim to mitigate risks and ensure patient safety during the vaccination process.^3^

## Conflict of Interest

The authors declare no conflict of interest.
